# Polyphenol-Enriched Extracts of *Prunus spinosa* Fruits: Anti-Inflammatory and Antioxidant Effects in Human Immune Cells Ex Vivo in Relation to Phytochemical Profile

**DOI:** 10.3390/molecules27051691

**Published:** 2022-03-04

**Authors:** Anna Magiera, Monika Ewa Czerwińska, Aleksandra Owczarek, Anna Marchelak, Sebastian Granica, Monika Anna Olszewska

**Affiliations:** 1 Department of Pharmacognosy, Faculty of Pharmacy, Medical University of Lodz, 1 Muszynskiego St., 90-151 Lodz, Poland; aleksandra.owczarek@umed.lodz.pl (A.O.); anna.marchelak@umed.lodz.pl (A.M.); monika.olszewska@umed.lodz.pl (M.A.O.); 2 Department of Biochemistry and Pharmacogenomics, Medical University of Warsaw, 1 Banacha St., 02-097 Warsaw, Poland; monika.czerwinska@wum.edu.pl; 3 Centre for Preclinical Research, Medical University of Warsaw, 1B Banacha St., 02-097 Warsaw, Poland; 4 Microbiota Lab, Centre for Preclinical Studies, Department of Pharmacognosy and Molecular Basis of Phytotherapy, Medical University of Warsaw, 1 Banacha St., 02-097 Warsaw, Poland; sgranica@wum.edu.pl

**Keywords:** blackthorn fruits, LC-MS, phenolic compounds, oxidative stress, inflammation, functional foods

## Abstract

The fresh fruits of *Prunus spinosa* L., a wild plum species, are traditionally used for dietary purposes and medicinal applications in disorders related to inflammation and oxidative stress. This study aimed to investigate the phytochemical composition of the fruits in the function of fractionated extraction and evaluate the biological potential of the extracts as functional products in two models of human immune cells ex vivo. Fifty-seven phenolic components were identified in the extracts by UHPLC-PDA-ESI-MS^3^, including twenty-eight new for the analysed fruits. Fractionation enabled the enrichment of polyphenols in the extracts up to 126.5 mg gallic acid equivalents/g dw total contents, 91.3 mg/g phenolic acids (caffeoyl-, coumaroyl-, and feruloylquinic acids), 41.1 mg/g flavonoids (mostly quercetin mono-, di- and triglycosides), 44.5 mg/g condensed proanthocyanidins, and 9.2 mg/g anthocyanins (cyanidin and peonidin glycosides). The hydroalcoholic extract and phenolic-enriched fractions of the fruits revealed significant ability to modulate pro-oxidant, pro-inflammatory, and anti-inflammatory functions of human neutrophils and peripheral blood mononuclear cells (PBMCs): they strongly downregulated the release of reactive oxygen species, TNF-*α*, and neutrophils elastase, upregulated the secretion of IL-10, and slightly inhibited the production of IL-8 and IL-6 in the cells stimulated by *f*MLP, *f*MLP+cytochalasin B, and LPS, depending on the test. Correlation studies and experiments on the pure compounds indicated a significant contribution of polyphenols to these effects. Moreover, cellular safety was confirmed for the extracts by flow cytometry in a wide range of concentrations. The results support the traditional use of fresh blackthorn fruits in inflammatory disorders and indicate extracts that are most promising for functional applications.

## 1. Introduction

A lot of epidemiological data provide solid evidence for the health benefits of diets rich in fruits and vegetables, mainly due to their bioactive constituents, including polyphenols, exhibiting the well-documented potential to resolve the interconnected pathological processes of inflammation and oxidative stress [1,2]. The essential hallmark of inflammation is the infiltration and hyperactivation of the primary immune cells, such as neutrophils and mononuclear cells (monocytes/macrophages) in the tissue site and the production of inflammatory mediators, including cytokines (interleukins, IL; tumour necrosis factor *α*, TNF-*α*), proteolytic enzymes (elastase, ELA-2), growth factors, arachidonic acid metabolites, and a vast diversity of reactive oxygen species (ROS), which collectively contribute to tissue dysfunction [3]. Failure to control this process is linked to many acute and chronic human disorders, such as bowel diseases, atherosclerosis, rheumatoid arthritis, and kidney diseases [3,4]. The conventional treatment for these health problems involves synthetic drugs, which often have emerging side effects. Therefore, global emphasis is on searching for plant-based foods as alternative options to prevent chronic inflammation with dietary intervention [5].

In the search of foods with anti-inflammatory activity, increased attention has been recently paid to the fruit of *Prunus spinosa* L. (blackthorn, sloe). It is a small rosaceous tree occurring primarily in south-central Europe but extending to the Scandinavian Peninsula, western Asia, and north-western Africa. The plant has also been introduced and locally naturalised in New Zealand, Tasmania, and North America, and is one of the most popular wild *Prunus* species worldwide [6]. The blackthorn fruit is a small, spherical, bluish-black drupe with a purple-blue frostlike bloom and yellow-greenish pulp. When fresh, the sloe plums have an astringent flavour; thus, they are harvested after being mellowed by frost and preferably consumed after processing into jams, jellies, juices, and alcoholic beverages, including wines, liqueurs, and tinctures [7].

In many countries, blackthorn fruit is a traditional herbal remedy recommended to treat inflammation-related disorders within the gastrointestinal and urinary tracts, respiratory system, and topically in oral and pharyngeal mucosa inflammation. Moreover, the fruit is used to treat diarrhoea, metabolic diseases (including diabetes and obesity) and as a heart-strengthening and anti-hypertensive agent [8,9,10]. It is believed that polyphenols are active components of the fruits, including anthocyanins, phenolic acids, and flavonoids [11,12,13,14,15,16,17]. However, the chemical composition of sloes is still insufficiently recognised as the reported fruit profiles are significantly less complex as expected compared to other organs of the same plant, such as flowers, leaves and stems [18,19,20].

The previous research on the biological activity of *P. spinosa* fruits has focused mainly on their antioxidant potential [11,14,21,22], with some data available on the antidiabetic, antimicrobial, anti-inflammatory and anticancer activity [15,17,23,24,25]. The accumulated results suggest the high value of blackthorn fruits as health-promoting food and source material for the production of functional supplements, but further research is required to characterise the underlying mechanisms, which might be relevant to their in vivo effects. To date, the antioxidant activity of the fruits has been studied in vitro mainly using simple chemical, non-cellular tests, such as the DPPH, ABTS, FRAP, and TBARS tests [11,14,21,22]. The cellular antioxidant potential has been demonstrated only for sloe juice in human malignant promonocytes (U937 cells) exposed to H_2_O_2_, with increased cellular viability being observed in the presence of the juice [21]. In the case of anti-inflammatory effects, Sabatini et al. [24] reported that the ethanol extract from blackthorn fruits could upregulate miR-126 and miR-146a expression levels and therefore downregulate the expression of the pro-inflammatory cytokine IL-6 and adhesion molecules ICAM-1 and VCAM-1 in lipopolysaccharide (LPS)-stimulated human endothelial cells (HUVEC). However, no information is available on the impact of blackthorn fruits on pro-inflammatory and pro-oxidant functions of normal human immune cells, which are critical factors in the inflammatory processes in vivo.

Several previous studies suggested that some technological treatments, such as fractionation [26] or loading in nanoparticles [25], might increase the anti-inflammatory effects of crude extracts from different fruits. In addition, fractionation might also improve the effectiveness of phytochemical profiling of the fruit extracts and studies on the contribution of particular compounds to their anti-inflammatory and antioxidant activity [26].

Therefore, the aim of the present study was (a) to characterise in details the polyphenolic profile of the fresh blackthorn fruits in the function of fractionated extraction; (b) to investigate the antioxidant and anti-inflammatory activity of the fruit extracts in human immune cells ex vivo, including neutrophils and peripheral blood mononuclear cells (PBMCs); (c) to select extracts that are most promising for their potential use as anti-inflammatory agents and functional products; and (d) to identify compounds responsible for the observed biological effects. A wide range of analytical techniques (UHPLC- PDA-ESI-MS^3^, HPLC-PDA, and UV-spectrophotometric methods) and authentic standards isolated from *P. spinosa* were used for phytochemical profiling to enable in-depth characterisation of secondary fruit metabolites. Moreover, the release of a series of pro-oxidant, pro-inflammatory and anti-inflammatory factors (ROS, IL-8, IL-6, TNF-*α*, ELA-2 and IL-10) was monitored during the cellular tests and the effects of the extracts were compared to those of a panel of positive standards including native blackthorn polyphenols. Furthermore, the cellular safety of the extracts was evaluated by flow cytometry.

## 2. Results

### 2.1. LC-PDA-ESI-MS^3^ Metabolite Profiling

**Figure 1:**
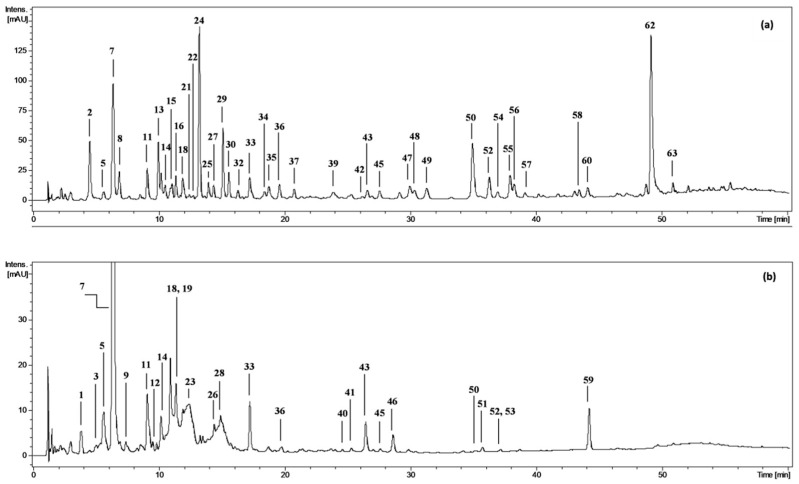
Representative UHPLC chromatograms at 280 nm of (**a**) diethyl ether fraction from fresh fruits of *P. spinosa*, DEFF; and (**b**) *n*-butanol fraction from fresh fruits, BFF. Peak numbers refer to those implemented in the Supplementary Table S1.

The phenolic profile of the fractionated extracts from fresh *P. spinosa* fruits was investigated by LC-PDA-ESI-MS^3^. The analytes were structurally characterised based on comparing their chromatographic and spectral properties with the literature data or reference standards, both commercial and isolated in our laboratory from flowers and leaves of *P. spinosa* ([18] and references therein). The enrichment of the phenolic fractions by fractionation of the source methanol-water (75:25, *v*/*v*) extract (MEF) with solvents of different polarity (diethyl ether, ethyl acetate, *n*-butanol, and water) allowed for the detection of 63 peaks in total (Figure 1, peaks **1**–**63**; Table S1) and full or tentative identification of 57 phenolic constituents, including 28 analytes found for the first time in sloes (the primary structures are shown in Figure S1). The identified compounds belonged to three main classes of polyphenols: (a) phenolic acids and aldehydes, (b) flavonols, and (c) anthocyanins.

#### 2.1.1. Phenolic Acids and Aldehydes

The leading and most structurally diversified group of polyphenols in sloes were phenolic acids (28 analytes; peaks: **1**, **2**, **5**–**16**, **18**, **20**–**22**, **24**, **27**, **29**–**35**, **39**, **59**) with UV-Vis absorption maxima in the range of 250–325 nm. Eight representatives of this group were identified by comparison with standards as protocatechuic (**2**), *p*-hydroxybenzoic (**8**), vanillic (**13**), caffeic (**16**), *p*-coumaric (**30**), and isomeric chlorogenic acids (**7**, **14**, **18**). The MS/MS data of compound **5** was similar to that of 3-*O*-caffeoylquinic acid (**7**), but its retention time was somewhat shorter; according to Jaiswal, Sovdat, Vivan and Kuhnert [27], it was identified as the *cis*-isomer of **7**. Compounds **6** and **9** revealed deprotonated molecular ions [M–H]^−^ at *m*/*z* 515 and MS^2^ base peaks at *m*/*z* 353 [M–H–hexose moiety]^−^, whose further fragmentation was consistent with that of **7**; thus, they were tentatively assigned as 3-*O*-caffeoylquinic acid hexosides [28]. According to the literature hierarchical discrimination keys [27,29], the peaks showing parent [M–H]^−^ ions at *m*/*z* 335, 337 and 367 were identified as isomeric caffeoylshikimic acids (**29**, **33**, **35**), *p*-coumaroylquinic acids (**11**, **21**, **27**) and feruloylquinic acids (**15**, **22**, **31**), respectively. Likewise, compound **39** with [M–H]^−^ at *m*/*z* 319 and a fragment ion at *m*/*z* 163 [*p*-coumaric acid–H]^−^ was identified as *p*-coumaroylshikimic acid [30]. The analytes **32** and **34** (*m*/*z* 501), due to the presence of the MS^2^ base peaks at *m*/*z* 223, typical for sinapic acid derivatives, and the neutral losses of 162 Da and 116 Da, diagnostic for a malate hexose moiety [31], were identified as sinapoyl malate hexosides. Analogously, compound **10**, with fragments at *m*/*z* 329 [M–H–116]^−^ and 167 [M–H–116–162]^−^ was identified as vanillic acid malate hexoside. Some other compounds representing phenolic acids (**1**, **12**, **20**) also revealed the loss of a hexose moiety ([M–162–H]^−^). Among them, compounds **12** and **20** with fragment ions at *m*/*z* 179 [caffeic acid–H]^−^ and 135 [caffeic acid–H–CO_2_]^−^ were identified as isomeric caffeic acid 3/4-*O*-hexosides. Likewise, peak **1** showed a fragment ion at *m*/*z* 167 [vanillic acid–H]^−^, which suggested the presence of vanillic acid *O*-hexoside [32]. In addition to phenolic acids, one phenolic aldehyde (vanillin, **24**) was detected and identified with standard.

#### 2.1.2. Flavonoids

The second group of polyphenols in sloes was formed by flavonols, revealing two UV-Vis absorption maxima at 270 and 350–370 nm. Their MS spectra in a negative ion mode showed the presence of fragment ions at *m*/*z* 301, 285, and 315, typical for quercetin (**62**), kaempferol and isorhamnetin aglycones, respectively. Flavonol mono-, di- and triglycosides (**36**, **38**, **40**–**58**, **60**–**61**) were discriminated based on the number and character of neutral losses of hexose (−162 Da), rhamnose (−146 Da), and pentose (−132 Da) moieties. Among them, nine quercetin glycosides were identified with standards as hyperoside (**42**), rutin (**44**), isoquercitrin (**45**), quercetin 3-*O*-(2″-*O*-*β*-d-glucopyranosyl)-*α*-l-arabino-furanoside (**46**), reinutrin (**47**), guaiaverin (**48**), avicularin (**50**), multinoside A (**51**), and quercitrin (**52**). Five other quercetin glycosides (**49**, **55**–**57**, **61**) fragmented with the neutral losses of malyl (−116 Da) or acetyl (−42 Da) moieties and were identified as the appropriate esters.

#### 2.1.3. Anthocyanins

Only four anthocyanins—glycosides of cyanidin (**19**, **23**) and peonidin (**26**, **28**)—with different sugar moieties were detected in the fruit extracts. Three compounds were identified with standards: **19** as cyanidin 3-*O*-*β*-d-glucopyranoside (CYG), **23** as cyanidin-3-*O*-rutinoside and **26** as peonidin-3-*O*-*β*-d-glucopyranoside. Peak **28**, showing the UV-Vis absorption maxima at 280 and 515 nm, typical for anthocyanins, and MS spectra characteristic for peonidin rhamnohexosides with a [M–H]^−^ ion at *m*/*z* 607 and a fragment ion at *m*/*z* 299 (aglycone moiety), resulting from the neutral loss of 308 Da ([M–132–162–H]^−^), was assigned as peonidin 3-*O*-rutinoside [33].

### 2.2. Quantitative Profile of the Extracts/Fractions

**Figure 2:**
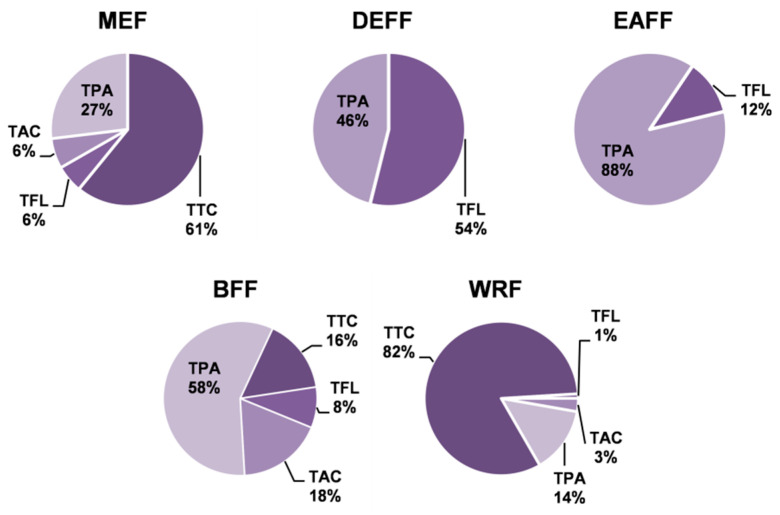
Contribution of individual groups of compounds to total phenolic contents in *P. spinosa* fruit extracts. TPA, TAC, and TFL: total contents of phenolic acids, anthocyanins, and flavonoids, respectively, determined by HPLC-PDA; TTC: total content of condensed tannins in procyanidin B2 (PB2) equivalents. For extracts codes, see Table 1.

The quantitative profiles of the fruit extracts/fractions differed depending on the extraction solvent (Table 1). The total phenolic content varied in the range of 64.6–126.5 mg GAE/g dw (TPC, determined by the Folin–Ciocalteu method) and 6.1–104.0 mg/g dw (TPH, determined by HPLC-PDA) with the highest values observed for DEFF and EAFF fractions. In all extracts/fractions, the TPC levels surpassed the TPH values, which represent the sum of low-molecular-weight polyphenols, including total flavonoids (TFL), total anthocyanins (TAC) and total phenolic acids (TPA). This gap came primarily from the presence of high-molecular-weight tannin-type proanthocyanidins (TTC). Figure 2 presents the relative ratio of particular families of polyphenols to the TPH+TTC levels that reflect the actual total phenolic level of the blackthorn extracts. The results indicated that the main constituents of MEF (Table 1, Figure 2) and thereby the primary polyphenols of fresh fruits (Table S2) were tannins, followed by phenolic acids, anthocyanins and flavonoids. Partitioning of MEF between solvents of different polarities led to the concentration of anthocyanins in BFF, flavonoids mainly in DEFF, phenolic acids in DEFF, EAFF and BFF, and tannins in WRF.

The TPA levels varied between 4.8 and 91.3 mg/g dw with the peak values in EAFF, where derivatives of phenolic acids prevailed (constituted 88% of the TPH+TTC contents). Pseudodepsides of quinic and shikimic acids (in both free and glycosylated forms), especially chlorogenic acids (**5**, **7**, **14**, **18**), were the most abundant compounds forming 47–96% TPAs. Neochlorogenic acid (**7**) dominated among the individual analytes with levels up to 15.6 mg/g dw in MEF and 49.6 mg/g dw in EAFF. Apart from pseudodepsides, the fruits contained simple cinnamic and benzoic acids (free and glycosylated). Their levels in the source extract were below the quantitation limits, but they were concentrated in DEFF, where protocatechuic acid (**2**) and vanillic acid (**13**) formed 24% of TPAs.

Anthocyanins are the primary pigments of fresh sloes. High levels of anthocyanins were revealed in MEF (4.6 mg/g dw) and after fractionation in BFF (9.2 mg/g dw). With the contents up to 42–49% of the TAC values, the dominant compound was CYG.

The levels of flavonoids were 0.3–41.1 mg/g dw, with the peak values in DEFF, where TFLs formed 50% TPH+TTCs. The profile of individual flavonoids varied significantly among particular extracts/fractions. Quercetin (QU) dominated in DEFF (formed 67% TFL), whereas glycosides prevailed in other fractions: avicularin (quercetin 3-*O*-*α*-l-arabinofuranoside, **50**) in EAFF (24% TFL) and rutin (**44**) in BFF (67% TFL). Avicularin and rutin were also the major flavonoids of the source extract MEF.

### 2.3. Influence on Cells Viability

The viability of neutrophils and PBMCs after 24–48 h of incubation with the fruit extracts/fractions and reference compounds was 85.0–93.4%, except for DEFF at 50–100 µg/mL, for which the cells’ viability was 72.4–82.8%. For the LPS-stimulated and control (non-stimulated) cells, this value was 91.8–96.4% and 92.9–94.4%, respectively (Figure S2).

### 2.4. Antioxidant Effect: Influence on ROS Production by Neutrophils

**Figure 3:**
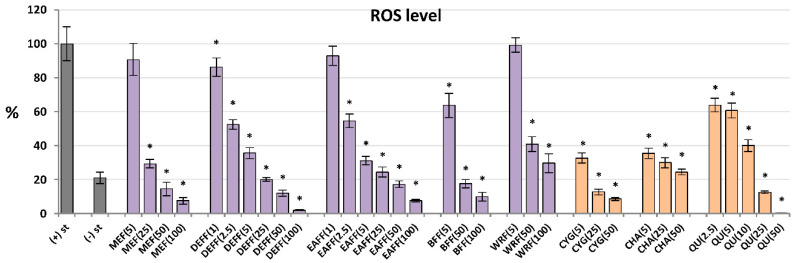
Effect of fruit extracts/fractions (1–100 µg/mL) and standards (5–50 µM) on the release of ROS, reactive oxygen species; Positive controls: QU, quercetin. For extracts codes see Table 1. Data expressed as means ± SD of five independent experiments performed with cells isolated from five independent donors. Statistical significance in Dunnett’s test: * *p* < 0.05 compared with the stimulated control.

For antioxidant activity testing, ROS were produced by cells after stimulation by *f*MLP. As shown in Figure 3, all analysed extracts/fractions revealed significant and dose-dependent antioxidant effects and reduced the oxidative burst in stimulated human neutrophils by at least 70% at 100 μg/mL. The most potent activity was observed for fractions enriched in phenolic acids and flavonoids (DEFF and EAFF), which downregulated the ROS release by at least 93% at 100 μg/mL and 43% at 2.5 μg/mL. The source extract MEFF effectively inhibited the ROS production at the levels 25–100 μg/mL (*p* < 0.05). For instance, at 50 μg/mL, it reduced the ROS release to 15%. This allowed for reaching the physiological value of ROS generation, which in non-stimulated cells was 21%. Among the reference compounds, the strongest antioxidant effects were revealed for CYG and chlorogenic acid (CHA), followed by QU (a positive standard), which downregulated the ROS production already at 5 μM (1.5–2.5 μg/mL).

### 2.5. Inhibition of ELA-2 Release by Neutrophils

**Figure 4:**
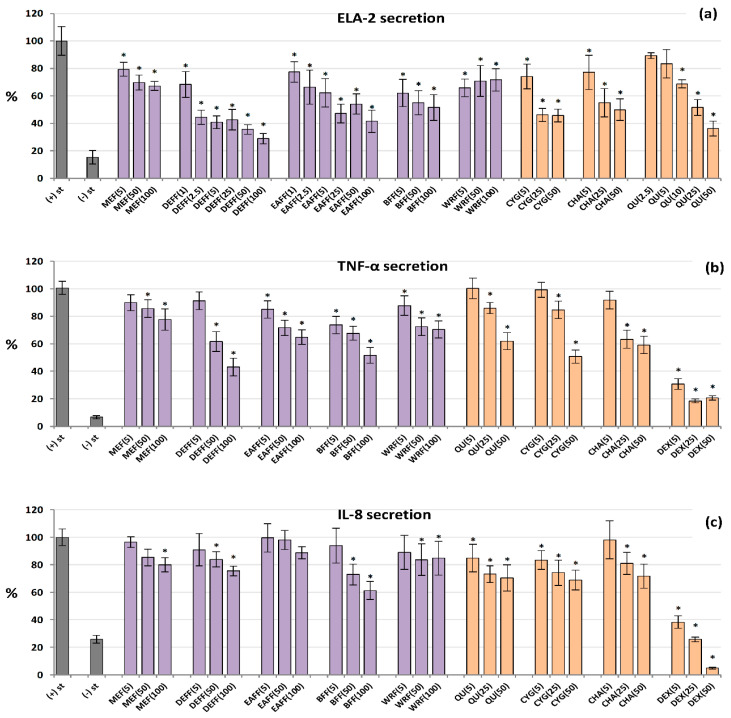
Effect of fruit extracts/fractions (1–100 µg/mL) and standards (5–50 µM) on pro-inflammatory functions of stimulated human neutrophils: effects on the release of (**a**) ELA-2, neutrophils elastase; (**b**) TNF-*α*, tumour necrosis factor *α*; and (**c**) IL-8, interleukin 8. Positive controls: QU, quercetin; DEX, dexamethasone. For extracts codes see Table 1. Data expressed as means ± SD of five independent experiments performed with cells isolated from five independent donors. Statistical significance in Dunnett’s test: * *p* < 0.05 compared with the stimulated control.

All of the tested extracts/fractions and reference compounds effectively inhibited the secretion of ELA-2 from neutrophils stimulated by *f*MLP+cytochalasin B, and in general their effects depended on the concentration (Figure 4a). The polyphenol-rich fractions (DEFF, EAFF, BFF), CYG, CHA and QU (a positive standard) reduced the enzyme release most strongly, by up to 71% at 100 μg/mL for DEFF, compared to control cells (untreated by the extracts). These analytes were also active at a low concentration of 5 μg/mL, and some of them even at 1–2.5 μg/mL (*p* < 0.05). For example, BFF reduced the ELA-2 secretion by 38% at 5 μg/mL. The source hydroalcoholic extract was less effective, but able to decrease the enzyme release by about 33% at 100 μg/mL.

### 2.6. Inhibition of IL-8 and TNF-α Production by Human Neutrophils

As shown in Figure 4b, all analysed extracts/fractions inhibited the secretion of TNF-*α* from LPS-stimulated neutrophils at 50–100 µg/mL (*p* < 0.05). The highest reduction (by up to 57%) was recorded for DEFF. At the lowest levels (5 μg/mL), BFF was most effective and downregulated the production of this cytokine by 26%. A similar range of activity was demonstrated for pure polyphenols. The effects on IL-8 release (Figure 4c) were weak and statistically significant only for selected extracts at 50–100 µg/mL, especially for BFF, which reduced the IL-8 levels by up to 39% at 100 µg/mL. Among pure polyphenols, CYG and QU were the most effective. Dexamethasone (DEX), a model anti-inflammatory drug (5–50 µM), significantly decreased the release of both TNF-*α* and IL-8.

### 2.7. Influence on TNF-α, IL-6 and IL-10 Production by Human PBMCs

**Figure 5:**
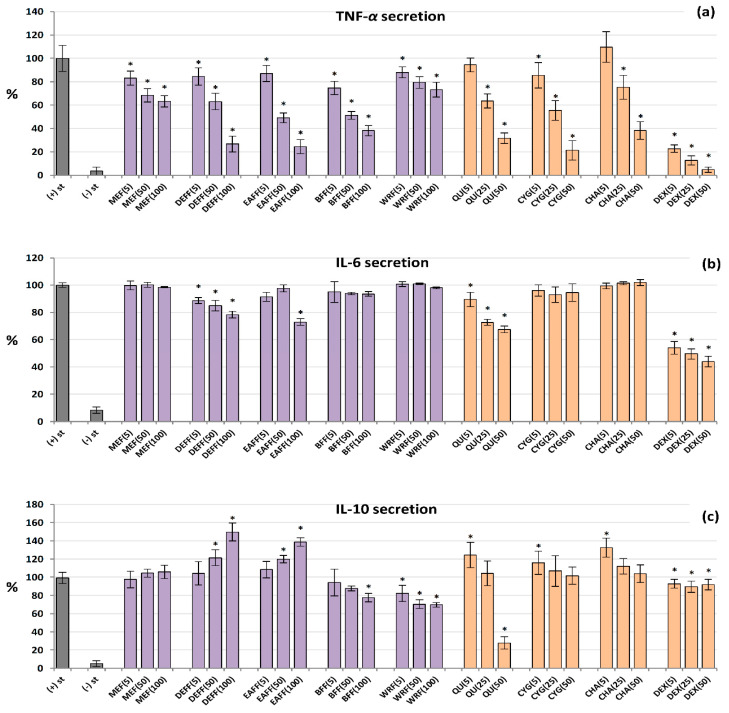
Effect of fruit extracts/fractions (5–100 µg/mL) and standards (5–50 µM) on pro-inflammatory and anti-inflammatory functions of stimulated human PBMCs: effects on the secretion of (**a**) TNF-*α*, tumour necrosis factor *α*; (**b**) IL-6 interleukin 6; and (**c**) IL-10, interleukin 10. Positive control: DEX, dexamethasone. For extracts codes see Table 1. Data expressed as means ± SD of five independent experiments performed with cells isolated from five independent donors. Statistical significance in Dunnett’s test: * *p* < 0.05 compared with the stimulated control.

The analysed extracts/fractions and pure polyphenols downregulated the release of TNF-*α* from LPS-stimulated PBMCs in a dose-dependent manner, and their effects were stronger than those revealed in the neutrophil model (Figure 5a). All of the extracts/fractions were effective even at 5 μg/mL (*p* < 0.05), and the most active were fractions enriched in anthocyanins, phenolic acids and flavonoids (BFF, DEFF and EAFF). For instance, EAFF reduced the TNF-*α* level by 16% at 5 μg/mL and 75% at 100 μg/mL, compared to LPS-stimulated control cells (*p* < 0.05). Similarly, all pure polyphenols also showed remarkable anti-TNF-*α* effects. In contrast, the effects on IL-6 secretion (Figure 5b) were statistically significant only for QU, DEFF and EAFF, which decreased the IL-6 levels by up to 33% at 100 µg/mL (*p* < 0.05). Concerning IL-10, the blackthorn extracts/fractions varied significantly in the ability to modulate the production of this cytokine (Figure 5c). In general, MEF did not influence its secretion (*p* > 0.05 in the entire concentration range), DEFF and EAFF stimulated its release, whereas BFF and WRF decreased its production (at 50–100 µg/mL). The stimulatory effect reached a maximum (50% increase) for DEFF and the inhibitory effect (32% decrease) for WRF at 100 µg/mL. Interestingly, pure polyphenols upregulated the IL-10 release at 5 µM, but their effects were insignificant at higher concentrations, except for QU, which strongly reduced the IL-10 levels at 50 µM. The positive control DEX significantly reduced the levels of both TNF-*α* and IL-6 but did not modulate the release of IL-10.

## 3. Discussion

Edible fruits are among the best sources of dietary polyphenols, and their regular intake is connected with the prevention and slowed development of oxidative stress and inflammation-related chronic human disorders [1,2]. For dietary intervention, fruits may be consumed fresh or processed into specialised functional products, including standardised extracts, which exhibit the most potent biological effects due to the increased content of polyphenols [34]. In this context, this work focused on the fruits of a wild European plum species—*P. spinosa*—and investigated their chemical profile as well as antioxidant and anti-inflammatory activity in the function of fractionated extraction. This is the first detailed phytochemical and bioactivity study of blackthorn fruits and the first comprehensive phytochemical survey of sloes harvested in central Europe (Poland).

Based on preliminary experiments and previous studies on sloes [11,12,15,16,22], for phytochemical analysis the fresh fruits were first extracted with methanol–water (75:25, *v*/*v*) to achieve high recovery of the target phenolic compounds. In accordance with the profiles reported for the fruits from other parts of Europe [12,13,16,17], the presence of four main polyphenolic classes, including phenolic acids, anthocyanins, flavonols and flavanols (condensed proanthocyanidins), was revealed (Table 1, Tables S1 and S2). However, thanks to the fractionation of crude hydroalcoholic extract and enrichment of the fractions in selected analytes, we were able to describe the composition of sloes in more detail and fully or tentatively identify 57 polyphenols, among which 28 compounds, mainly flavonoids and phenolic acids, were found for the first time (Figure 1a,b; Table S1). In consequence, the fruit profile appeared similarly complex as that of blackthorn flowers (57 identified constituents according to Marchelak et al. [18]) and significantly more diversified than stems and leaves of the plant (25 and 21 identified components, respectively [19,20]). In comparison to other plant parts, the blackthorn fruits may be distinguished by the presence of anthocyanins and lack of low-molecular-weight flavanols. Nevertheless, high and comparable complexity of the polyphenolic fractions of fruits and flowers might explain the popularity of both plant materials in traditional phytotherapy [8,9,10].

The TPC levels in sloes were in good agreement with some previous reports, both in the case of dry extract MEF (87.57 mg GAE/g dw versus 83.40 mg GAE/g dw [11]) and fresh fruits (13.69 mg GAE/g fw versus 17.69 mg GAE/g fw [15]). The total level of low-molecular-weight phenols (TPH) in fresh fruits (4.46 mg/g; Table S2) was higher than the literature values (2.29–3.37 mg/g fw; [16,22,24]), probably because of more detailed profiling. Moreover, a large fraction of condensed proanthocyanidins was detected (6.96 mg PCB2/g fw) that previously has been quantified in sloes at significantly lower levels (1.85 mg GAE/g fw; [16]). Recently, Popović et al. [17] suggested that the variability of polyphenolic profiles in sloes harvested in different geographic areas might be due to changing climatic and genetic factors, and the relative ratio between different classes of polyphenols might be especially prone to change. Indeed, we observed the shares of anthocyanins and phenolic acids to the TPH levels (16.2 and 68.9%, respectively) similar to those found earlier for the fruits collected in colder parts of Europe [17] but significantly different from those revealed in the south of the continent [16,22,24], where anthocyanins (50–70%) clearly prevailed over phenolic acids (15–32%). Nevertheless, as the TPC of fresh sloes (1.37 g GAE/100 g fw; Table S2) surpassed the literature levels in fruits valued for their health-promoting properties and anti-inflammatory effects in humans, such as cranberries (0.45 g/100 g), blueberries (0.13 g/100 g) and chokeberries (about 1.01 g/100 g) [35], the present study confirms the significant potential of blackthorn fruits for industrial and functional applications.

Ethnopharmacological sources suggest that blackthorn fruits may control systemic and local inflammation, especially within the digestive tract, but also in the urinary tract and cardiovascular system [8,9,10]. Recent preclinical and epidemiological studies of polyphenol-rich fruits of similar composition, such as cherries or blueberries, have confirmed their exceptional effectiveness against gastrointestinal inflammation [36,37,38]. Those effects might be connected with the oral bioavailability profile of polyphenols. It is well established that the absorption of polyphenols in the upper digestive tract is relatively moderate and results in at most micromolar (1–5 µM) phenolic levels in the bloodstream, while 90–95% of the bolus reaches the colon and accumulates in the millimolar range, making the activity most dramatic for gastrointestinal disorders [39]. In the case of primary *P. spinosa* polyphenols, their millimolar levels (taking into account their molecular masses) might range from 130–180 µg/mL for phenolic acids to 500–700 µg/mL for glycosides and pseudodepsides (Table S1). Therefore, the antioxidant and anti-inflammatory activity of the blackthorn extracts in the models of human immune cells was tested during the present study in a wide range of concentrations (1–100 µg/mL). This range was a good compromise between the cells’ viability (Figure S2) and the polyphenolic levels that might be achieved in plasma (1–5 µg/mL) and in local compartments of the digestive tract (25–100 µg/mL) after oral administration of the fruits or extracts. 

Neutrophil migration and massive infiltration into the colon mucosa and lamina propria is a hallmark of numerous inflammatory disorders of the digestive tract, for example inflammatory bowel disease (IBD) [40]. The excessive or prolonged activation of neutrophils promotes an increased mobilisation of monocytes differentiating into macrophages that are attracted to the damaged tissues where they extend inflammatory reactions [3]. The present study revealed that the extracts from fresh *P. spinosa* fruits significantly and in a dose-dependent manner modulate the pro-oxidant and pro-inflammatory functions of human neutrophils and monocytes (PBMCs) ex vivo with the most spectacular effects towards the release of ROS, TNF-*α*, ELA-2 and IL-10.

Oxidative stress is a crucial feature of digestive tract inflammation. The chief cause of oxidative stress in IBD is the overproduction of ROS such as ONOO^−^, NO^•^, O_2_^•−^, H_2_O_2_, HO^•^ and HClO by neutrophils in the process of oxidative burst, which cause progressive damage of the intestinal mucosa and stimulate tissue inflammation [5]. We observed that the blackthorn extracts significantly inhibited the oxidative burst of stimulated neutrophils, and the ROS release correlated negatively with the phenolic levels, both TPC and TPH+TTC (*r*_max_ = −0.7398, *p* < 0.001; Table S3). As the polyphenol-rich fractions (DEFF, EAFF, BFF) strongly reduced the ROS production at even 2.5–5 µg/mL, and their activity was up to 50-fold stronger than that of phenolic-poor WRF, the polyphenolic constituents might be considered primarily responsible for these effects. This was confirmed by the potent activity of pure blackthorn polyphenols CYG and CHA compared to the positive control.

The cellular antioxidant effects of polyphenols may be related to their direct ROS-scavenging potential or to some indirect mechanisms, including the regulation of transcription factors, such as NF-κB, and the secretion of regulatory cytokines, including TNF-*α*, which can prime neutrophils for oxidative burst [26]. Indeed, Fraternale et al. [21] showed that sloe juice exhibits cytoprotective activity and significantly increases the viability of H_2_O_2_-treated human promonocytes U937, probably due to H_2_O_2_ scavenging. Recently, we have reported that polyphenols of *P. spinosa* flowers are able to effectively scavenge several other in vivo-relevant ROS, such as O_2_^•−^, ONOO^−^, HO^•^ and HClO [41]. On the other hand, Sabatini et al. [24] and Tiboni et al. [25] have demonstrated that the ethanol extracts from sloes decrease the expression of interleukin-1 receptor-associated kinase (IRAK-1) in stimulated U937 cells, which plays a crucial role in regulating signalling cascades and NF-κB activation. In the present study, we observed that the sloe extracts significantly diminished the release of TNF-*α* from both neutrophils and PBMCs. TNF-*α* is a master cytokine of the NF-κB pathway and potent priming agonist of immune cells that orchestrate the inflammatory response and stimulate the release of numerous pro-inflammatory mediators, including ROS [42]. TNF-*α* is also a key cytokine in the pathogenesis of IBD and a therapeutic target in modern anti-inflammatory therapies [40]. It is worth noting that the blackthorn extracts were especially effective downregulators of the TNF-*α* secretion in PBMCs, the leading producers of this cytokine in vivo [42]. Polyphenols contributed primarily to the anti-TNF-*α* effects as a significant negative correlation was found between the phenolic levels (TPC or TPH+TTC) and TNF-*α* release in PBMCs (*r*_max_ = −0.8689, *p* < 0.001; Table S3).

An important finding of the present study is also the potent ability of the blackthorn extracts to inhibit the secretion of ELA-2 from stimulated neutrophils. This enzyme is a serine protease that breaks the extracellular matrix by degrading its main components, such as elastin and collagen [26]. In IBD patients, ELA-2 increases tissue permeability, potentiates the inflammatory response, impairs mucosal regeneration, and is closely associated with disease progression [43]. The blackthorn polyphenols seem essential for the release inhibition of ELA-2, but because the relationship between their levels (TPC or TPH+TTC) and the enzyme secretion was relatively weak, although significant (*r*_max_ = −0.4481, *p* < 0.05; Table S3), the synergistic effects of non-phenolic components of sloes should be taken into consideration. Nevertheless, strong anti-TNF-*α*, anti-ROS and anti-ELA-2 effects of the extracts might partly explain the traditional indication of sloes in gastrointestinal inflammation, and the TPC level might be recommended as a simple quality marker of the extracts for their functional application.

ROS, TNF-*α* and ELA-2 appeared to be the main targets for the sloe extracts, but the influence on the secretion of IL-10 might also be relevant. IL-10 is the primary anti-inflammatory cytokine in humans that inhibits the release of pro-inflammatory cytokines and attenuates the inflammatory process in the mucosa [3]. Therefore, intense stimulation of the IL-10 production by DEFF and EAFF might be beneficial in the context of their future use. Interestingly, other fractionated extracts (BFF and WRF) exhibited the opposite effects. This fact might suggest that low-molecular-weight blackthorn polyphenols (TPH), enriched in DEFF and EAFF, indeed stimulate this cytokine, while non-phenolic compounds or TTC (enriched in hydrophilic fractions) inhibit its secretion, but further investigation is required to confirm this hypothesis.

The previous studies on sloes concluded that anthocyanins are mainly responsible for the anti-inflammatory and cellular antioxidant activity of the fruits [21,24,25]. This might indeed be true for fruits collected in Mediterranean regions as anthocyanins dominated in their phenolic fractions [16,22,24]. However, the impact of other polyphenols, especially phenolic acids and flavonoids, should not be overlooked. As evidenced by our results, the fractions rich in these types of polyphenols and anthocyanin-free (DEFF and EAFF) had stronger or comparable effects to the anthocyanin-rich BFF. Hence, it might be considered that phenolic acids are primary active components of sloes collected in continental climate regions such as Poland, where it seems they prevailed over anthocyanins, but other polyphenols have an additive or synergistic impact.

## 4. Materials and Methods

### 4.1. Plant Material

Fruit samples of *P. spinosa* L. in the phase of full ripeness (Figure S3) were collected in October 2018 by hand picking from shrubs naturally growing in Krasnobród (50°32′41″ N and 23°12′55″ E), Poland (near the Roztocze National Park). The plant material was authenticated by prof. M.A. Olszewska according to Popescu and Caudullo [6]. The voucher specimen (no. KFG/HB/18006-PSP-FRUIT) was deposited in the herbarium of the Department of Pharmacognosy, Medical University in Lodz, Poland. After collecting, the fresh fruits were immediately and directly frozen and stored at −15 °C until use.

### 4.2. Extracts Preparation

A sample of fresh fruits (1008.0 g) was poured with a small amount of heated methanol–water (75:25, *v*/*v*), ground in a blender and refluxed three times with methanol-water (75:25, *v*/*v*; 1.5 L and 20 min each). The solvent was filtered off and the filtrates were combined to give the source extract MEF (157.6 g dw). Next, the sample of MEF (145.8 g dw) was suspended in warm (40 °C) water (1.5 L) and subjected to sequential liquid–liquid extraction with organic solvents (10 × 200 mL each) to yield diethyl ether fraction (DEFF, 1.6 g dw), ethyl acetate fraction (EAFF, 1.8 g dw), *n*-butanol fraction (BFF, 9.2 g dw) and water residue (WRD, 9.7 g dw). The organic solvent extracts were dried by vacuum evaporation (Rotavapor R-200, Büchi^®^, Flawil, Switzerland) and the water-containing fractions were lyophilised (Alpha 1–2/LD Plus freeze dryer; Christ, Osterode, Germany). All solvents were of analytical grade (Avantor Performance Materials, Gliwice, Poland).

### 4.3. Phytochemical Profiling

#### 4.3.1. Qualitative LC-MS/MS Profiling

The LC-PDA-ESI-MS^3^ analysis was performed on a UHPLC-3000 RS system (Dionex, Dreieich, Germany) coupled with a diode array detector (Dionex) and an AmaZon SL ion trap mass spectrometer with an ESI interface (Bruker Daltonik, Bremen, Germany). Separations were carried out on a Kinetex XB-C18 column (1.7 μm, 150 mm × 2.1 mm i.d.; Phenomenex, Torrance, CA, USA) at 25 °C. The mobile phase consisted of solvent A (water/formic acid, 100:0.1, *v*/*v*) and solvent B (acetonitrile) with the elution profile as follows: 0–10 min, 6–13% B (*v*/*v*); 10–15 min, 13% B (*v*/*v*); 15–19 min, 13–15% B (*v*/*v*); 19–28 min, 15% B (*v*/*v*); 28–44 min, 15–23% B (*v*/*v*); 44–55 min, 23–40% B (*v*/*v*); 55–60 min, 40% B (*v*/*v*); 60–65 min, 40–6% B (*v*/*v*); 65–80 min, 6% B (*v*/*v*). All gradients were linear. The flow rate was 0.3 mL/min. Before injection, sample extract solutions (10.0 mg/1.5 mL) were filtered through a PTFE syringe filter (13 mm, 0.2 μm, Whatman, Pittsburgh, PA, USA). UV-Vis spectra were recorded over a range of 200–600 nm. The LC eluate was introduced directly into the ESI interface without splitting and analysed in both negative and positive ion modes. ESI parameters: the nebulizer pressure was 40 psi; dry gas flow 9 L/min; dry temperature 300 °C; and capillary voltage 4.5 kV. MS^2^ and MS^3^ fragmentations were obtained in Auto MS/MS mode for the most abundant ions at the time. Analysis was carried out scanning from m/z 200 to 2200. Identification of the analytes was confirmed by comparing the retention times and spectra (UV-Vis, MS/MS) with standards and/or literature data. All solvents were of HPLC grade (Avantor Performance Materials, Gliwice, Poland). For reference standards, see Supplementary S.2.

#### 4.3.2. Quantitative Profiling

The total contents of polyphenols (TPC) and condensed tannins (proanthocyanidins, TTC) were determined spectrophotometrically by Folin–Ciocalteu and vanillin assay, as described previously by Marchelak et al. [18] and Sun et al. [44], respectively. The results were expressed as equivalents of HPLC-grade standards (Sigma-Aldrich, St. Louis, MO, USA) of gallic acid (GAE) and procyanidin B2 (PB2) per dw of the extracts. 

The quantitative HPLC-PDA analyses were performed on a Shimadzu Prominence-i LC-2030C 3D chromatograph (Shimadzu, Kyoto, Japan) according to the method validated previously for the fingerprint analysis of the flower extracts of *P. spinosa* [45], modified slightly for the study of the fruit matrix. Separations were carried out on a C18 Ascentis^®^ Express column (2.7 μm, 150 mm × 4.6 mm; Supelco, Bellefonte, PA, USA) with a C18 Ascentis^®^ 2.7 Micron Guard Cartridge (2.7 μm, 5 mm × 4.6 mm; Supelco). Samples of the tested extracts (2.5–20 mg) were dissolved in 10 mL of methanol–water (75:25, *v*/*v*), filtered through a polytetrafluoroethylene (PTFE) syringe filter (25 mm, 0.2 µm, Ahlstrom, Helsinki, Finland), and the filtrate was directly injected (5 µL) into the HPLC system. The elution system consisted of solvent A (0.5% water solution of orthophosphoric acid, *w*/*v*), solvent B (acetonitrile) and solvent C (tetrahydrofuran). The elution profile is presented in Table 2. The flow rate was 1.0 mL/min, the column was maintained at 25 °C, and the injection volume was 5 μL.

The UV-Vis spectra were recorded over a range of 190–550 nm. The wavelength for detection and quantification was set to 250 nm for hydroxybenzoic acid derivatives, 325 nm for caffeic acid derivatives, 310 nm for other hydroxycinnamic acid derivatives, 350 nm for flavonol glycosides, 350 nm for flavonol aglycones, and 520 nm for anthocyanins. The peak purity was assessed based on the degree of similarity of the UV spectra across the peak over the whole data collection range using a LabSolutions software (Shimadzu, Kyoto, Japan). Calibration was performed with the use of 24 authentic standards and the method was re-validated according to Marchelak et al. [18]. The validation parameters for 7 fruit-specific standards (not involved in the previous study) are shown in Table 3. The analytes were quantified as equivalents of external standards per dw of the extracts, depending on their structures and UV-Vis spectra: protocatechuic acid for hydroxybenzoic acid derivatives, CHA for monocaffeoylquinic acid isomers, cynarin for dicaffeoylquinic acid isomers, caffeic acid or *p*-coumaric acid for other hydroxycinnamic acid derivatives; isoquercitrin for flavonoid monoglycosides, rutin for flavonoid diglycosides, and CYG for anthocyanins. All quantitative results were recalculated for the fresh weight (fw) of fruits using the extraction yield of MEF.

### 4.4. Antioxidant and Anti-Inflammatory Activity in Cellular Models

#### 4.4.1. Isolation of Human Neutrophils and PBMCs

The buffy coat fractions (a by-product of blood fractionation for transfusions) were obtained from the Warsaw Blood Donation Centre, where they were collected from adult male donors (18–35 years old). The donors were non-smokers, were clinically recognised to be healthy, and did not take any medications. The study conformed to the principles of the Declaration of Helsinki. As it involved commercially available biological material, it did not require approval of a bioethics committee.

Neutrophils were isolated by dextran sedimentation, hypotonic lysis of erythrocytes and, centrifugation in a Ficoll–Hypaque gradient (PAA Laboratories, Pasching, Austria) according to Michel et al. [46]. PBMCs were isolated by a Ficoll–Hypaque gradient centrifugation as described previously [47]. After isolation, the cells were suspended in RPMI 1640 culture medium, (Ca^2+^)-free phosphate-buffered saline (PBS; Thermo Fisher Scientific, Waltham, MA, USA), or (Ca^2+^)-free Hanks’ balanced salt solution (HBSS; Sigma-Aldrich, St. Louis, MO, USA), and were maintained at 4 °C before use.

#### 4.4.2. Neutrophils and PBMCs Viability

The potential cytotoxicity of the extracts, fractions and pure compounds (analytes) was examined by standard flow cytometry (BD FACSCalibur, BD Biosciences, San Jose, CA, USA) with propidium iodide (PI) staining and Triton X-100 solution as a positive control [46]. The analytes were tested at the final concentrations of 1–100 µg/mL for the extracts/fractions and 5–50 µM for pure compounds. The analyses were performed after 24h of incubation for neutrophils, and after 48h of incubation for PBMCs.

#### 4.4.3. ROS Generation by Human Neutrophils

The inhibition of ROS production was analysed by the luminol-dependent chemiluminescence method after *N*-formyl-l-methionyl-l-leucyl-l-phenylalanine (*f*-MLP) stimulation [46]. The changes in chemiluminescence were measured in 96-well plates over a 40 min period at intervals of 2 min using a microplate reader (Synergy 4, BioTek, Winooski, VT, USA). The percentage of ROS secretion was calculated in comparison to the control untreated by the investigated analytes. Quercetin (5–50 µM) was used as a positive control. All reagents were from Sigma-Aldrich (St. Louis, MO, USA).

#### 4.4.4. ELA-2 Release by Human Neutrophils

The ELA-2 secretion by *f*-MLP-cytochalasin B-stimulated neutrophils was determined using *N*-succinyl-alanine-alanine-valine-*p*-nitroanilide (SAAVNA) as a substrate according to Michel et al. [46] with some modifications. Briefly, 200 μL of cell suspension (4 × 10^6^/mL) in HBSS was pre-incubated in 96-well plates for 30 min at 37 °C with 5% CO_2_ in the presence of an analyte (50 μL) dissolved in HBSS. The final levels of the analytes were the same as those used in the viability tests. The liberated *p*-nitrophenol was measured at 412 nm over a period of 300 min with 20 min intervals using a microplate reader (SYNERGY 4, BioTek, Winooski, VT, USA). The percentage of the ELA-2 release was calculated in comparison to the control untreated by the investigated analytes. Quercetin (5–50 µM) was used as a positive control. All reagents were from Sigma-Aldrich (St. Louis, MO, USA).

#### 4.4.5. Secretion of IL-8, IL-6, TNF-*α* and IL-10 by Immune Cells

The release of pro- and anti-inflammatory cytokines by LPS-stimulated cells was analysed as described earlier [46,47], with slight modifications. Briefly, the working cell concentration was 2 × 10^6^/mL for neutrophils and 4 × 10^6^/mL for PBMCs, and the incubation time was 24 h for analyte-treated neutrophils and 48 h for analyte-treated PBMCs. The final levels of the analytes were the same as those used in the viability tests. Dexamethasone (5–50 µM) was applied as a positive control. LPS (from Escherichia coli O111:B4) was purchased from Merck Millipore (Billerica, MA, USA), and all other reagents from Sigma-Aldrich (St. Louis, MO, USA).

### 4.5. Statistics and Data Analysis

Results were expressed as means ± standard deviation (SD) for replicate determinations. The statistical analyses (calculation of SD, one-way analysis of variance, HSD Tukey’s HSD test, Dunnett’s test) were carried out using the Statistica13.1Pl software for Windows (StatSoft, Krakow, Poland) at a significance level of *α* = 0.05.

## 5. Conclusions

This is the first report on the antioxidant and anti-inflammatory activity of fresh fruits of *P. spinosa* in normal human immune cells. With 57 identified constituents (28 new for the fruits), the study also introduces significant novelty to the phytochemistry of sloes. The results indicate that fresh blackthorn fruits from Poland (continental climate) accumulate high contents of polyphenols, mainly condensed proanthocyanidins and phenolic acids, followed by anthocyanins and flavonols. As evidenced by correlation studies, polyphenols significantly contribute to the antioxidant and anti-inflammatory effects of the fruit extracts. The biological effects of the extracts are significant and most pronounced for the release of some key pro-inflammatory (ROS, TNF-*α*, ELA-2) and anti-inflammatory (IL-10) factors, which are down- and upregulated, respectively, in immune cells. Considering the fact that native polyphenols accumulate to a large extent in the intestines, these observations might explain the traditional application of blackthorn fruits in chronic inflammatory diseases within the gastrointestinal tract. The study also documents the role of fractionation for effective phytochemical profiling and concentration of active fruit polyphenols. Due to the superior phenolic contents and activity parameters, the DEFF, EAFF and BFF appear to be the most advantageous for functional applications. Nonetheless, further studies of their activity mechanisms, potential toxicity and in vivo effects, including intestinal metabolism, are required to verify their health benefits.

## Figures and Tables

**Table 1:** Quantitative profile of the *P. spinosa* fruit extracts (mg/g dw).

	MEF	DEFF	EAFF	BFF	WRF
**Total contents:**					
TPC (GAE)	87.57 ± 3.54 ^b^	126.49 ± 1.41 ^a^	123.63 ± 3.68 ^a^	68.23 ± 0.12 ^c^	64.59 ± 0.61 ^c^
TPH	28.56 ± 0.58 ^d^	81.83 ± 0.80 ^b^	104.02 ± 1.92 ^a^	43.17 ± 1.14 ^c^	6.07 ± 0.14 ^e^
TPA	19.67 ± 0.33 ^d^	35.15 ± 1.12 ^b^	91.26 ± 2.16 ^a^	29.62 ± 1.10 ^c^	4.79 ± 0.08 ^e^
TAC	4.64 ± 0.11 ^b^	n.d.	n.d.	9.17 ± 0.33 ^a^	0.96 ± 0.06 ^c^
TFL	4.25 ± 0.21 ^c^	41.11 ± 0.41 ^a^	12.21 ± 0.32 ^b^	4.38 ± 0.17 ^c^	0.32 ± 0.02 ^d^
TTC (PB2)	44.53 ± 1.93 ^a^	n.d.	n.d.	8.02 ± 0.23 ^c^	28.36 ± 0.41 ^b^
**Individual compounds:**					
Avicularin (**50**)	1.32 ± 0.14 ^c^	2.52 ± 0.02 ^b^	2.98 ± 0.14 ^a^	0.41 ± 0.06 ^d^	n.d.
Guaiaverin (**48**)	n.d.	1.05 ± 0.05 ^a^	0.61 ± 0.06 ^b^	n.d.	n.d.
Hyperoside (**42**)	0.11 ± 0.005 ^c^	0.94 ± 0.07 ^b^	1.51 ± 0.03 ^a^	n.d.	n.d.
Isoquercitrin (**45**)	0.09 ± 0.004 ^c^	0.21 ± 0.01 ^b^	0.97 ± 0.07 ^a^	0.12 ± 0.01 ^c^	n.d.
Reinutrin (**47**)	n.d.	0.38 ± 0.02 ^a^	0.24 ± 0.02 ^b^	n.d.	n.d.
Rutin (**44**)	1.60 ± 0.02 ^c^	0.29 ± 0.04 ^d^	2.25 ± 0.27 ^b^	2.93 ± 0.11 ^a^	0.22 ± 0.03 ^d^
Quercitrin (**52**)	0.15 ± 0.01 ^c,d^	1.66 ± 0.04 ^b^	1.91 ± 0.11 ^a^	0.13 ± 0.01 ^d^	n.d.
Quercetin (**62**, QU)	n.d.	27.45 ± 0.09 ^a^	1.25 ± 0.13 ^b^	n.d.	n.d.
Cyanidin 3-*O*-glucoside (**19**, CYG)	1.96 ± 0.12 ^b^	n.d.	n.d.	4.46 ± 0.12 ^a^	0.55 ± 0.06 ^c^
Cyanidin 3-*O*-rutinoside (**23**)	1.39 ± 0.07 ^b^	n.d.	n.d.	2.28 ± 0.10 ^a^	0.41 ± 0.05 ^c^
Peonidin-3-*O*-glucoside (**26**)	0.99 ± 0.04 ^b^	n.d.	n.d.	1.68 ± 0.07 ^a^	n.d.
Protocatechuic acid (**2**)	n.d.	5.22 ± 0.11 ^a^	0.63 ± 0.02 ^b^	n.d.	n.d.
*p*-Hydroxybenzoic acid (**8**)	n.d.	0.51 ± 0.06 ^a^	0.47 ± 0.01 ^a^	n.d.	n.d.
Vanillic acid (**13**)	n.d.	3.37 ± 0.19 ^a^	n.d.	n.d.	n.d.
*p*-Coumaric acid (**30**)	n.d.	0.52 ± 0.03 ^a^	n.d.	n.d.	n.d.
Neochlorogenic acid (**7**)	15.56 ± 0.30 ^c^	10.93 ± 0.84 ^d^	49.62 ± 1.51 ^a^	24.47 ± 1.07 ^b^	3.40 ± 0.15 ^e^
Chlorogenic acid (**14**, CHA)	0.94 ± 0.02 ^b^	0.62 ± 0.02 ^c^	4.75 ± 0.03 ^a^	0.39 ± 0.01 ^d^	n.d.
Cryptochlorogenic acid (**18**)	1.57 ± 0.07 ^d^	4.85 ± 0.10 ^b^	28.74 ± 0.37 ^a^	3.63 ± 0.12 ^c^	n.d.
Vanillin (**24**)	n.d.	5.57 ± 0.38 ^a^	0.55 ± 0.04 ^b^	n.d.	n.d.

Results are presented as means ± SD (*n* = 3). Numbers in parentheses (first column, in bold) refer to those in Figure 1 and Supplementary Table S1. For each parameter, different superscript letters (a–e) indicate significant differences (*p* < 0.05) in Tukey’s HSD test. MEF, methanol-water (75:25, *v*/*v*) extract of fresh fruits; DEFF, diethyl ether fraction of MEF; EAFF, ethyl acetate fraction of MEF; BFF, *n*-butanol fraction of MEF; WRF, water residue of MEF; TPC, total phenolic contents in gallic acid equivalents (GAE); TPH, total contents of low-molecular-weight phenols determined by HPLC-PDA; TPA, total phenolic acids; TAC, total anthocyanins; TFL, total flavonoids; TTC, total tannins in procyanidin B2 (PB2) equivalents. n.d.: below the limits of quantitation (LOQ) or detection (LOD).

**Table 2:** Elution profile for HPLC-PDA quantitation of the fruit polyphenols.

Solvent B (Acetonitrile)		Solvent C (Tetrahydrofuran)	
Time (min)	Concentration (%, *v*/*v*)	Time (min)	Concentration (%, *v*/*v*)
0–1	3.0 (isocratic elution)	0–8	2.5 (isocratic elution)
1–25	3.0 → 20.5 (linear gradient)	8–9	2.5 → 6.5 (linear gradient)
25–45	20.5 → 50.0 (linear gradient)	9–19	6.5 (isocratic elution)
45–50	50.0 (isocratic elution)	19–24	6.5 → 9.0 (linear gradient)
50–52	50.0 → 3.0 (return to initial condition)	24–50	9.0 (isocratic elution)
52–60	3.0 (equilibration)	50–52	9.0 → 2.5 (return to initial condition)
		52–60	2.5 (equilibration)

**Table 3:** Validation parameters for HPLC-PDA quantitative method.

Analyte	λ (nm)	Regression (Linear Model)		*r*	Linear Range (μg/mL)	*F*-Test	LOD (μg/mL)
		Equation	*n*				
Protocatechuic acid	260	y = 15884.45x	6	0.9998	0.64–63.7	19979.99	0.398
*p*-Hydroxybenzoic acid	260	y = 25596.21x	6	0.9997	0.63–62.8	13094.52	0.365
Vanillic acid	260	y = 16884.29x	6	0.9997	0.56–55.5	15385.86	0.373
Vanillin	280	y = 18673.22x	6	0.9999	0.67–66.8	32063.03	0.389
Cyanidin 3-*O*-*β*-d-glucopyranoside	520	y = 17908.10x	6	0.9990	0.52–52.3	4091.50	0.339
Cyanidin 3-*O*-rutinoside	520	y = 10946.63x	6	0.9997	0.51–51.0	12466.71	0.325
Peonidin 3-*O*-*β*-d-glucopyranoside	520	y = 12604.30x	6	0.9956	0.48–48.5	906.70	0.340

λ, detection wavelength; y, peak area; x, concentration of standard in (μg/mL); *n*, number of concentration levels (data points) used for construction of the regression equation; *F*-test, value of the statistical Fisher variance ratio for the experimental data.

## Data Availability

Not applicable.

## Supplementary Materials

The following supporting information can be downloaded online. S.1. Results: Table S1. UHPLC-PDA-ESI-MS^3^ identification data of compounds detected in the extracts from fresh fruits of *P. spinosa*; Table S2. Quantitative profile of the *P. spinosa* fruit (mg/g fw); Table S3. Correlation (*r*) coefficients and probability *p*-Values of linear relationships between antioxidant and anti-inflammatory activity parameters and phenolic contents of *P. spinosa* fruit extracts; Figure S1. Structures of the dominant polyphenolic compounds of *P. spinosa* fruits extracts; Figure S2. Effect of the *P. spinosa* fruit extracts and pure compounds on viability (membrane integrity) of human immune cells; S.2. Reference Standards; S.3. Plant Material: Figure S3. Representative image of a sample of fresh *P. spinosa* fruits. References [48,49,50,51] are cited in the supplementary materials.

## Sample Availability

Samples of the fruits and extracts are available from the authors.

## Abbreviations

BFF	*n*-butanol fraction of MEF
CHA	chlorogenic acid (5-*O*-caffeoylquinic acid)
CYG	cyanidin 3-*O*-*β*-d-glucopyranoside
DEFF	diethyl ether fraction of MEF
DEX	dexamethasone
dw	dry weight
EAFF	ethyl acetate fraction of MEF
ELA-2	elastase 2 (neutrophils elastase)
*f*MLP	*N*-formyl-l-methionyl-l-leucyl-l-phenylalanine
FRAP	ferric reducing antioxidant power
fw	fresh weight
GAE	gallic acid equivalents
IBD	inflammatory bowel disease
IL	interleukin
LOD	limits of detection
LOQ	limits of quantitation
LPS	bacterial lipopolysaccharide
MEF	methanol-water (75:25, *v*/*v*) extract of fresh fruits
PB2	procyanidin B2
PBMCs	peripheral blood mononuclear cells
QU	quercetin
ROS	reactive oxygen species
TAC	total content of anthocyanins
TFL	total content of flavonoids
TNF-*α*	tumour necrosis factor *α*
TPA	total content of phenolic acids
TPC	total phenolic content (Folin–Ciocalteu assay)
TPH	total phenolic content (sum of individual phenolics by HPLC)
TTC	total content of tannin-type proanthocyanidins
WRF	water residue of MEF after fractionated extraction
