# Supplementary material for: Polyphenol-Enriched Extracts of Prunus spinosa Fruits: Anti-Inflammatory and Antioxidant Effects in Human Immune Cells Ex Vivo in Relation to Phytochemical Profile
Source: Molecules. 2022 Mar 4;27(5):1691. doi: 10.3390/molecules27051691 (PMC8912089; doi:10.3390/molecules27051691)
Supplement: Supplementary file 1 [file molecules-27-01691-s001.zip › molecules-1619495-supplementary.pdf]

molecules

Supplementary Material for:

Polyphenol-Enriched Extracts of *Prunus spinosa* Fruits: Anti-Inflammatory and Antioxidant Effects in Human Immune Cells Ex Vivo in Relation to Phytochemical Profile

Anna Magiera [1,*], Monika Ewa Czerwińska[2,3], Aleksandra Owczarek[1], Anna Marchelak[1], Sebastian Granica[4] and Monika Anna Olszewska[1]

[1] Department of Pharmacognosy, Faculty of Pharmacy, Medical University of Lodz, 1 Muszynskiego St., 90-151 Lodz, Poland
[2] Department of Biochemistry and Pharmacogenomics, Medical University of Warsaw, 1 Banacha St., 02-097 Warsaw, Poland
[3] Centre for Preclinical Research, Medical University of Warsaw, 1B Banacha St., 02-097 Warsaw, Poland
[4] Microbiota Lab, Centre for Preclinical Studies, Department of Pharmacognosy and Molecular Basis of Phy-totherapy, Medical University of Warsaw, 1 Banacha St., 02-097 Warsaw, Poland
* Correspondence: anna.magiera@umed.lodz.pl (A. Magiera); Tel.: +48 503316997

Supplementary Material

Table of Contents

S.1. Results

Table S1. UHPLC-PDA-ESI-MS^3 identification data of compounds detected in the dry extracts from fresh and dried fruits of *P. spinosa*.

Figure S1. Structures of the dominant polyphenolic compounds of *P. spinosa* fruits extracts.

Table S2. Quantitative profile of the *P. spinosa* fruit (mg/g fw).

Figure S2. Effect of the fruit extracts and pure compounds on viability (membrane integrity) of human immune cells.

Table S3. Correlation (*r*) coefficients and probability (*p*) values of linear relationships between antioxidant and anti-inflammatory activity parameters and phenolic contents of *P. spinosa* fruits extracts.

S.2. Reference Standards

S.3. Plant Material

Figure S3. Representative image of a sample of fresh *P. spinosa* fruits.

References

S.1. Results

Table S1. UHPLC-PDA-ESI-MS[3] identification data of compounds detected in the extracts from fresh fruits of *P. spinosa*.

Peak	Analyte	*Rt* (min)	UV λ_{max} (nm)	[M–H]⁻ (m/z)	Fragmentary ions	[M–H]+ (m/z)	Fragmentary ions	Fruit extracts	References
1	Vanillic acid *O*-hexoside	3.9	250<br>290	329	167	-	-	BFF, EAFF, MEF, WRF	-
2	Protocatechuic acid[a,b]	4.6	260<br>295	153	153	155	155	DEFF, EAFF	-
3	Unidentified	5.2	325	517	335	-	-	BFF, MEF	-
4	Unidentified	5.2	275	315	179, 161	-	-	WRF	-
5	cis-3-*O*-Caffeoylquinic acid[b]	5.7	322	353	191, 179	-	-	EAFF, MEF, WRF	[1]
6	3-*O*-Caffeoylquinic acid hexoside[b]	6.2	325<br>295	515	353 (MS[3]: 191, 179), 335, 341, 179			BFF	[2]
7	3-*O*-Caffeoylquinic acid<br>(neochlorogenic acid)[a]	6.7	325<br>265<br>211	353	191, 179, 135	-	-	BFF, DEFF, EAFF, MEF, WRF	[3]
8	*p*-Hydroxybenzoic acid[a]	7.1	267<br>290	137	-	-	-	DEFF, EAFF	-
9	3-*O*-Caffeoylquinic acid hexoside[b]	7.8	210<br>325<br>270	515	353 (MS[3]: 191, 179), 191, 341	-	-	BFF, EAFF	[2]
10	Vanilloyl malate hexoside[b]	8.9	260	445	329, 167	-	-	EAFF	-
11	3-*O*-*p*-Coumaroylquinic acid	9.2	310	337	163, 191	-	-	BFF, DEFF, EAFF, MEF, WRF	[1]
12	Caffeic acid 3/4-*O*-hexoside	9.5	317<br>285	341	179, 135	-	-	BFF	-
13	Vanillic acid[a]	10.1	216<br>260<br>290	167	167	169	169	DEFF	-
14	5-*O*-Caffeoylquinic acid (chlorogenic acid, CHA)[a]	10.6	325	353	191, 179	-	-	BFF, DEFF, EAFF, MEF	[3]
15	*cis*-3-*O*-Feruloylquinic acid[b]	11.0	215<br>325<br>293	367	193	-	-	DEFF, EAFF	[1]
16	Caffeic acid[a]	11.2	323<br>214<br>286	179	-	-	-	DEFF	-
17	Unidentified	11.2	300<br>225	415	293	-	-	BFF	-
18	4-*O*-Caffeoylquinic acid<br>(cryptochlorogenic acid)[a]	11.5	325<br>215	353	173, 191, 179	-	-	BFF, DEFF, EAFF, MEF	[3]
19	Cyanidin 3-*O*-glucoside (CYG)[a]	11.6	515<br>280	447	285, 401	449	287	BFF, MEF, WRF	-

20	Caffeic acid 3/4-*O*-hexoside	12.1	320	341	179, 135	-	-	EAFF	-
21	4-*O*-*p*-Coumaroylquinic acid	12.3	310	337	173	-	-	DEFF	[1]
22	3-*O*-Feruloylquinic acid	12.5	322	367	193	-	-	DEFF, EAFF	[1]
23	Cyanidin 3-*O*-rutinoside[a]	12.6	515<br>280	593	285, 467	595	287	BFF, MEF, WRF	-
24	Vanillin[a,b]	13.3	285	-	-	153	153	DEFF, EAFF	-
25	Unidentified	14.1	216<br>262	295	251	-	-	DEFF	-
26	Peonidin 3-*O*-glucoside[a]	14.4	515<br>280	607	299	-	301	BFF, MEF	[4]
27	*cis*-3-*O*-*p*-Coumaroylquinic acid[b]	14.6	305	337	163	-	-	DEFF, EAFF	[1]
28	Peonidin 3-*O*-rutinoside	15.0	515<br>280	607	299	-	301	BFF, MEF, WRF	[4]
29	4-*O*-Caffeoylshikimic acid[b]	15.2	325	335	179, 135	-	-	DEFF, EAFF	[1]
30	*p*-Coumaric acid[a]	15.8	310	163	-	-	-	DEFF	-
31	4-*O*-Feruloylquinic acid[b]	16.5	320<br>217	367	176	-	-	EAFF	[1]
32	Sinapoyl malate hexoside[b]	16.5	280<br>313	501	223	-	-	DEFF	-
33	Caffeoylshikimic acid[b]	17.4	280	335	161, 135, 179	-	-	BFF, DEFF, EAFF, MEF, WRF	[1]
34	Sinapoyl malate hexoside[b]	18.5	275	501	223, 339	-	-	DEFF	[5]
35	Caffeoylshikimic acid[b]	18.9	215	335	161, 179	-	-	DEFF, EAFF	[1]
36	Quercetin dirhamnoside-hexoside[b]	19.7	350	755	301, 609	-	303	BFF	-
37	Unidentified	20.9	280	317	287	-	-	DEFF	-
38	Kaempferol dihexoside[b]	23.3	280<br>330	609	447, 285	-	287	MEF	-
39	*p*-Coumaroylshikimic acid[b]	23.8	300<br>205	319	119, 145, 275, 163, 257	-	-	DEFF, EAFF	[6]
40	Quercetin hexoside-pentoside	24.7	354	595	301, 433	-	303	BFF, MEF	-
41	Quercetin rhamnoside-hexoside	25.6	355	609	301	-	303	BFF, MEFF	-
42	Quercetin 3-*O*-*β*-D-galactoside (hyperoside)[a]	26.2	264<br>355	463	301	-	303	DEFF, EAFF, MEF	-
43	Kaempferol rhamoside-dihexoside	26.3	280<br>350	755	593, 285	-	287	WRF	-
44	Quercetin 3-*O*-(6″-*O*-*α*-L-rhamnopyranosyl)-*β*-D-glucopyranoside (rutin)[a]	26.6	265<br>350	609	301	-	303	BFF, DEFF, EAFF, MEF, WRF	-
45	Quercetin 3-*O*-*β*-D-glucopyranoside (isoquercitrin)[a]	27.7	260<br>355	463	301	-	303	BFF, DEFF, EAFF, MEF	-
46	Quercetin 3-*O*-(2″-*O*-*β*-D-glucopyranosyl)-*α*-L-arabinofuranoside[a,b]	28.7	255<br>355	595	433, 301	-	303	BFF, MEF, WRF	-
47	Quercetin 3-*O*-*α*-D-xylopyranoside (reinutrin)[a,b]	30.1	256<br>356	433	301	435	303	DEFF, EAFF	-
48	Quercetin 3-*O*-*α*-L-arabinopyranoside (guaiaverin)[a,b]	30.4	255<br>355	433	301	435	303	DEFF, EAFF	-

49	Quercetin acetyl-dihexoside[b]	31.5	266<br>350	667	301	-	303	DEFF	-
50	Quercetin 3-*O*-*α*-L-arabinofuranoside (avicularin)[a,b]	35.1	255<br>355	433	301	435	303	BFF, DEFF, EAFF, MEF	-
51	Quercetin 3-*O*-(4″-*O*-*β*-D-glucopyranosyl)-*α*-L-rhamnopyranoside (multinoside A)[a,b]	35.8	254<br>355	609	447, 301	611	449, 303	BFF, EAFF, MEF	-
52	Quercetin 3-*O*-*α*-L-rhamnopyranoside (quercitrin)[a]	36.5	255<br>355	447	301	449	303	BFF, DEFF, EAFF, MEF	-
53	Isorhamnetin rhamnoside-hexoside (Isorhamnetin rutinoside)	37.2	260<br>350	623	315	-	-	BFF, MEF	-
54	Isorhamnetin rhamnoside[b]	37.2	350	461	315	-	-	DEFF	-
55	Quercetin malyl-pentoside[b]	38.0	254<br>350	549	433, 301	-	435, 303	DEFF, EAFF	-
56	Quercetin acetyl-hexoside	38.4	250<br>350	505	301	-	303	DEFF, MEF	-
57	Quercetin malyl-pentoside[b]	39.3	255<br>355	549	433, 301	-	435, 303	DEFF	-
58	Quercetin pentoside	43.7	255<br>350	433	301	435	303	DEFF	-
59	Caffeoylquinic acid butyl ester[b]	44.2	325<br>292	409	161, 135, 191	-	-	BFF	[7]
60	Kaempferol hexoside[b]	44.4	255<br>355	447	285	-	287	DEFF	-
61	Quercetin acetyl-hexoside-rhamoside	46.5	254<br>350	651	609, 301	-	303	EAFF, MEF	-
62	Quercetin (QU)[a]	49.2	255<br>356	301	-	-	303	DEFF, EAFF	-
63	Unidentified	50.9	350	337	322	-	-	DEFF	-

[a] Identified with authentic standards. [b] Detected in *P. spinosa* fruits for the first time. *Rt*, retention times. UV λ_{max}, absorbance maxima in UV-Vis spectra. $[M–H]^-$, deprotonated molecular ions in MS spectra recorded in a negative ion mode. $[M+H]^+$, protonated molecular ions in MS spectra recorded in a positive ion mode. Nomenclature of quinic acid esters including chlorogenic acid isomers is according to IUPAC rules adopted by Clifford et al. [3,8].

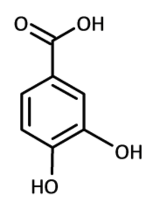

Protocatechuic acid **(2)**

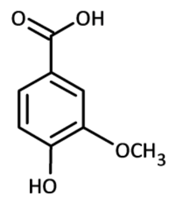

Vanillic acid **(13)**

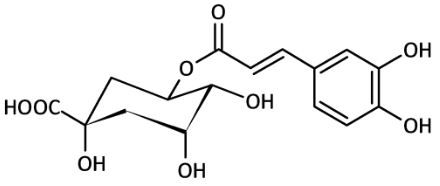

Chlorogenic acid **(14, CHA)**

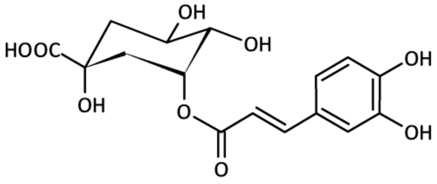

Neochlorogenic acid (**7**)

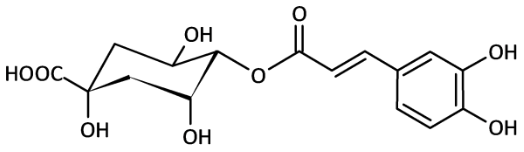

Cryptochlorogenic acid (**18**)

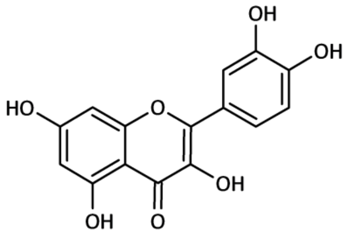

Quercetin (**62**, QU)

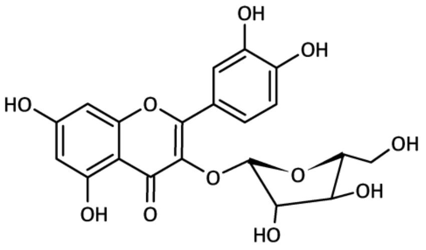

Avicularin (**50**)

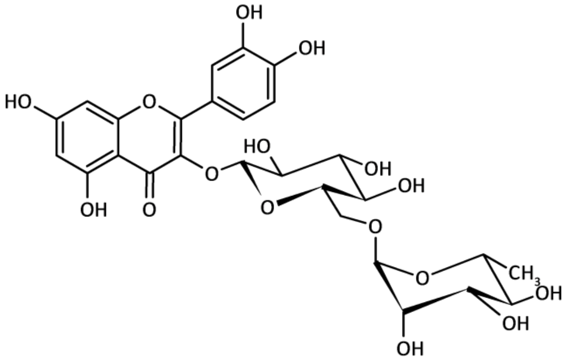

Rutin (**44**)

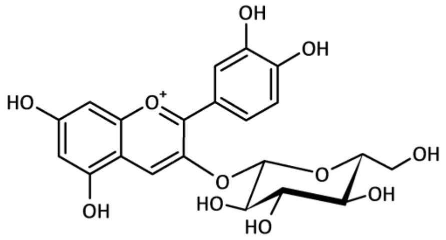

Cyanidin 3-*O*-glucoside (**19**, CYG)

Figure S1. Structures of the dominant polyphenolic compounds of *P. spinosa* fruits extracts. Numbers in parentheses (bold font) refer to those in Figure 1 (main article) and supplementary Table S1.

Table S2. Quantitative profile of the *P. spinosa* fruit (mg/g fw).

**Total contents**	
TPC (GAE)	13.69 ± 0.55
TPH	4.46 ± 0.09
TPA	3.07 ± 0.05
TAC	0.72 ± 0.02
TFL	0.66 ± 0.03
TTC (PB2)	6.96 ± 0.30
**Individual compounds**	
Avicularin (**50**)	0.21 ± 0.02
Hyperoside (**42**)	0.03 ± 0.0007
Isoquercitrin (**45**)	0.01 ± 0.0006
Rutin (**44**)	0.25 ± 0.003
Quercitrin (**52**)	0.02 ± 0.001
Cyanidin 3-O-glucoside (**19**, CYG)	0.31 ± 0.02
Cyanidin 3-O-rutinoside (**23**)	0.22 ± 0.01
Peonidin-3-O-glucoside (**26**)	0.15 ± 0.01
Neochlorogenic acid (**7**)	2.43 ± 0.05
Chlorogenic acid (**14**, CHA)	0.15 ± 0.003
Cryptochlorogenic acid (**18**)	0.24 ± 0. 01

Results are presented as means ± SD (n = 3). Numbers in parentheses (first column, in bold) refer to those in Figure 1 (main article) and supplementary Table S1. TPC, total phenolic contents in gallic acid equivalents (GAE); TPH, total contents of low-molecular-weight phenols determined by HPLC-PDA; TPA, total phenolic acids; TAC, total anthocyanins; TFL, total flavonoids; TTC, total tannins in procyanidin (PB2) B2 equivalents.

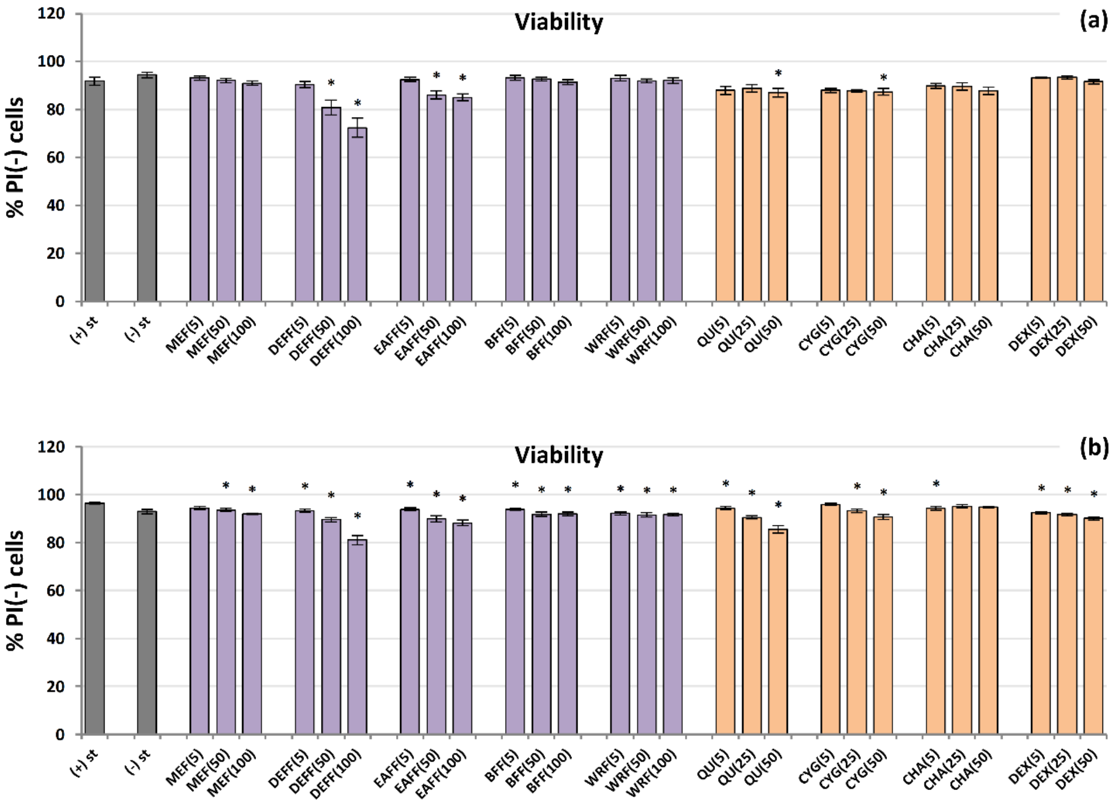

Figure S2. Effect of fruit extracts/fractions (5-100 μg/mL) and standards (5-50 μM) on viability (membrane integrity) of human immune cells expressed as a percentage of PI(-) cells (propidium iodide-negative): **(a)** effect on viability of neutrophils after 24 h incubation; **(b)** effect on viability of PBMCs after 48 h incubation. Standards: DEX, dexamethasone; QU, quercetin; CYG, cyanidin 3-*O*-glucoside; CHA, chlorogenic acid; Positive control: Triton X-100 solution (98.6% of PI(+) cells). Data expressed as means ± SD of five independent experiments performed with cells isolated from five independent donors. Statistical significance in Dunnett's test: *$p < 0.05$ compared with the stimulated control.

Table S3. Correlation (r) coefficients and probability (p) values of linear relationships between biological activity parameters and phenolic contents of *P. spinosa* fruits extracts.

Neutrophils				
**r (*p*) for**	**ROS level**	**TNF-α secretion**	**ELA-2 secretion**	**IL-8 secretion**
TPC	-0.7455 (0.000)*	-0.7463 (0.001)*	-0.4777 (0.029)*	-0.4545 (0.089)
TPH+TTC	-0.7398 (0.000)*	-0.6504 (0.009)*	-0.4481 (0.042)*	-0.3927 (0.148)
**PBMCs**				
**r (*p*) for**		**TNF-α secretion**	**IL-10 secretion**	**IL-6 secretion**
TPC		-0.8689 (0.000)*	0.5787 (0.024)*	-0.6200 (0.014)*
TPH+TTC		-0.8761 (0.000)*	0.6116 (0.015)*	-0.6070 (0.016)*

Values calculated using activity and concentration parameters reported in Table 1 and Figures 3-5. TPC, total phenolic contents in gallic acid equivalents (GAE); TPH, total contents of low-molecular-weight phenols determined by HPLC-PDA; TTC, total tannins in procyanidin (PB2) B2 equivalents. Asterisks mean statistical significance of the linear relationships (p < 0 05).

S.2. Reference Standards

All standards used for phytochemical profiling and biological activity tests were of HPLC grade (purity at least 95%). The standards of dexamethasone, protocatechuic acid, *p*-hydroxybenzoic acid, 3-*O*-caffeoylquinic acid (neochlorogenic acid), 4-*O*-caffeoylquinic acid (cryptochlorogenic acid), 5-*O*-caffeoylquinic acid (chlorogenic acid, CHA), cynarin, *p*-coumaric acid, caffeic acid, vanillic acid, vanillin, quercetin (QU), quercetin 3-*O*-*α*-L-arabinofuranoside (avicularin), quercetin 3-*O*-*β*-D-galactopyranoside (hyperoside), quercetin 3-*O*-*β*-D-glucopyranoside (isoquercitrin), quercetin 3-*O*-(6″-*O*-*α*-L-rhamnopyranosyl)-*β*-D-glucopyranoside (rutin) and (-)-epicatechin were purchased from Sigma-Aldrich (St. Louis, MO, USA). The standards of cyanidin 3-*O*-*β*-D-glucopyranoside (CYG), cyanidin 3-*O*-rutinoside and peonidin 3-*O*-*β*-D-glucopyranoside were purchased from PhytoLab (Vestenbergsgreuth, Germany). All other standards, such as quercetin 3-*O*-*α*-L-arabinopyranoside (guaiaverin), quercetin 3-*O*-*β*-D-xylopyranoside (reinutrin), quercetin 3-*O*-*α*-L-rhamnofuranoside (quercitrin), quercetin 3-*O*-(4″-*O*-*β*-D-glucopyranosyl)-*α*-L-rhamnopyranoside and quercetin 3-*O*-(2″-*O*-*β*-D-glucopyranosyl)-*α*-L-arabinofuranoside were isolated previously in our laboratory from flowers and leaves of *P. spinosa* with at least 95% HPLC purity [9,10].

S.3. Plant Material

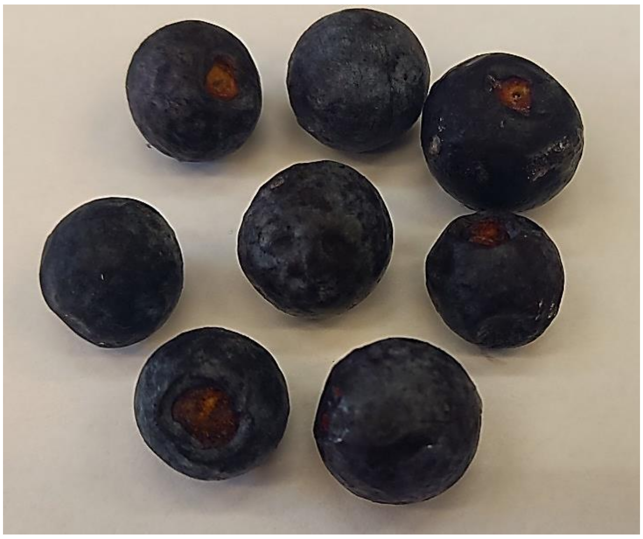

Figure S3. Representative image of a sample of fresh *P. spinosa* fruits.

References

1 Jaiswal, R.; Sovdat, T.; Vivan, F.; Kuhnert, N. Profiling and characterization by LC-MSn of the chlorogenic acids and hydroxycinnamoylshikimate esters in maté (*Ilex paraguariensis*). *J. Agri. Food Chem.* **2010**, *58*, 5471-5481.

2 Jaiswal, R.; Müller, H.; Müller, A.; Karar, M. G.; Kuhnert, N. Identification and characterization of chlorogenic acids, chlorogenic acid glycosides and flavonoids from *Lonicera henryi* L. (Caprifoliaceae) leaves by LC-MS^n. *Phytochemistry* **2014**, *108*, 252–263.

3 Clifford, M.N.; Johnston, K.L.; Knight, S.; Kuhnert, N. Hierarchical scheme for LC-MS^n identification of chlorogenic acids. *J. Agri. Food Chem.* **2003**, *51*, 2900-2911.

4 Colombo, F.; Di Lorenzo, C.; Regazzoni, L.; Fumagalli, M.; Sangiovanni, E.; de Sousa, L.P.; Bavaresco, L.; Tomasi, D.; Bosso, A.; Aldini, G.; Restani, P.; Dell'Agli, M. Phenolic profiles and anti-inflammatory activities of sixteen table grape (*Vitis vinifera* L.) varieties. *Food Funct.* **2019**, *10*, 1797.

5 Morreel, K.; Saeys, Y.; Dima, O.; Lu, F.; Van de Peer, Y.; Vanholme, R.; Ralph, J.; Vanholme, B.; Boerjan, W. Systematic structural characterization of metabolites in Arabidopsis via candidate substrate-product pair networks. *Plant Cell* **2014**, *26*, 929–945.

6 Ben Said, R.; Hamed, A.I.; Mahalel, U.A.; Al-Ayed, A.S.; Kowalczyk, M.; Moldoch, J.; Oleszek, W.; Stochmal, A. Tentative Characterization of Polyphenolic Compounds in the Male Flowers of *Phoenix dactylifera* by Liquid Chromatography Coupled with Mass Spectrometry and DFT. *Int. J. Mol. Sci.* **2017**, **18**, E512.

7 Wu, Z.J.; Ma, X.L.; Fang, D.M.; Qi, H,Y.; Ren, W.J.; Zhang, G.L. Analysis of caffeic acid derivatives from *Osmanthus yunnanensis* using electrospray ionization quadrupole time-of-flight mass spectrometry. *Eur. J. Mass Spectrom.* **2009**, *15*, 415–429.

8 Clifford, M.N.; Knight, S.; Kuhnert, N. Discriminating between the six isomers of dicaffeoylquinic acid by LC-MS^n. *J. Agri. Food Chem.* **2005**, *53*, 3821–3832.

9 Olszewska, M.; Wolbiś, M. Further flavonoids from the flowers of *Prunus spinosa* L. *Acta Pol. Pharm.* **2002a**, *59*, 133-137.

10 Olszewska, M.; Wolbiś, M. Flavonoids from the leaves of *Prunus spinosa* L. *Pol. J. Chem.* **2002b**, *76*, 967–974.
